# Pre-hospital advanced airway management in children: a challenge that training can handle

**DOI:** 10.1186/s13049-017-0432-7

**Published:** 2017-12-04

**Authors:** Joanna B. Watterson, Cliff Reid, Brian J. Burns, Luke Regan

**Affiliations:** 1 Greater Sydney Area Helicopter Emergency Medical Service (GSA-HEMS), New South Wales Ambulance, Sydney, NSW Australia; 2 New York University School of Medicine, New York, NY USA; 3 Discipline of Emergency Medicine, Sydney Medical School, University of Sydney, Camperdown, NSW Australia; 4 Emergency Department, Raigmore Hospital, Inverness, Scotland UK

**Keywords:** Paediatric intubation, Out-of-hospital paediatric intubation, Training, PHETI

## Abstract

We respond to the Tarpgaard et al. article reporting on pre-hospital endotracheal intubation (PHETI) success and complications by Danish critical care teams including critical care anaesthetists. We compare the authors’ results with previously published results from our service’s experience with PHETI in a similar patient population, also with physician and paramedic medical teams. From 25 children <16 years of age, the Danish study reports overall success, and first-pass success, and complication rates of 96, 75 and 20%, respectively. A recently published study of 82 patients that we completed revealed the following results: 100, 91 and 14%, respectively. We propose training and operating protocols we believe contribute to this relative success in paediatric PHETI.

## To the editor,

Tarpgaard et al. published a prospective descriptive study designed to assess pre-hosptial paediatric endotracheal intubation success by critical care anaesthetists [1]. We appreciate the authors’ recognition of the importance of this issue and the need for more research in this area. To this end, we applaud their publishing of success and complication rates for this rare but definitive procedure. However, we reject the authors’ suggestion that paediatric patients represent a substantial advanced airway management challenge. We present data to support our argument that, given a training environment characterized by structured simulation and use of standard operating procedures, paediatric advanced airway management is no more challenging than adult advanced airway management in the out-of-hospital environment.

The 2015 Danish study reported an overall success rate of 96% (24/25), a first pass success-rate of 75% (18/24) and a complication rate of 20% (5/25). In a forthcoming 2017 study in *Annals of Emergency Medicine*, we reported on the same end points in a similar but larger (*n* = 82) study population [2]. Overall success rate was 100% (82/82), first-look success rate was 91% (75/82) and complication rate per attempt was 14% (13/90).

The patient populations and systematic factors of these two studies are comparable in many ways, especially scarcity of paediatric PHETI as a potential obstacle to proficiency, indication for advanced airway management, medications used for Rapid Sequence Intubation (RSI), age range of patients, and definitions of complications. Furthermore, the study designs were similar, with near-identical primary and secondary outcomes, and both were potentially subject to errors due to low population size, as well as registration and recall bias. Tarpgaard et al. acknowledge that the subgroup of patients <2 may have been sicker than their older counterparts. Similarly, our study reported a higher frequency of cardiac and respiratory arrest in our youngest patients.

However, there are some differences between the two studies worth noting. Tarpgaard et al. report on a younger population, with the majority of their patients <2 years of age. Our patient population reflected a higher percentage with trauma, and a lower percentage with pre-existing disease. Another key difference is the exclusion of interhospital missions by their study and inclusion in ours, in which interhospital missions represented 24% of the total. Additionally, paramedics in our service routinely perform intubation, whereas in the Danish study, Emergency Medical Technician team members never intubate [3]. The physicians in our study included not only anaesthetists, but also emergency physicians.

One issue in comparing the results of these studies directly is that our study does not directly compare paediatric and all-population PHETI success. However, we previously published a study on difficult intubation factors that offers success metrics for all PHETI that can be compared [4]. Unlike the Danish study, we did not find a lower first pass success rate in paediatric patients (Table 1). The lower success and higher complication rates in the adult study are likely reflective of improved training and greater protocolization of ETI in our service in recent years, rather than age-related differences in performance. We believe this in light of trends in yet-unpublished data we use to track airway management performance.

**Table 1:** Comparison of paediatric and all-population PHETI success

Article	Tarpgaard et al. [1]	Tarpgaard et al. [1]	Burns, Watterson et al. [2]	Burns, Habig et al. [4]
Age	Paediatric <16	All	Paediatric <16	All
N	25	735	82	443
Study Period	2/2011–11/2012	2/2011–11/2012	1/2010–4/2015	9/2009–9/2013
Overall success rate	96%	99.7%	100%	98.9%
First-attempt success rate	75%	77.6%	91%	84.0%
Complication Rate	20%	13.9%	14%	26.2%

While the adult and paediatric studies from our service have different study periods and are not directly comparable, we believe that our results demonstrate that paediatric intubation need not be considered a substantial challenge. High overall and first-attempt success, as well as low complication rate, are achievable. It is worth noting that complication rates remained lower even though our definition of a critical complication, desaturation/hypoxia, was more stringent, at oxygen saturation of <93%, than that of Tarpgaard et al., at <90%.

Ours is not the only service that has recently documented paediatric PHETI success. Schmidt et al. retrospectively reviewed records of 225 patients <17 and found a first-attempt success rate of 95.3% and an overall success rate of 98.6% [5]. Eich et al. designed a prospective observation study of 52 patients <15, documenting 85% first-attempt success rate and 98% overall success [6].

There are a number of factors we believe contribute to our relative success in PHETI in general. These include mandatory use of the service Standard Operating Procedure (SOP), RSI manual and challenge-response checklist for pre-hospital RSI. These materials are routinely integrated into training of service clinicians before and during employment. All aspects of pre-hosptial RSI are standardized and drilled in training, including a team-based approach which involves both the physician and paramedic in the intubation process, specific pharmacological agents and dosages, patient positioning to optimize view on laryngoscopy, use of boguie, and routine ‘thirty second drills’ used to improve sub-optimal glottic visualization. For paediatric patients, clinicians are taught to use paediatric reference cards providing drug dosing, tube sizing and insertion depth based on patient weight or age. We believe that sufficient training and preparation can make airway management challenges feasible, even in the complex prehospital environment. This is evidenced by our finding that, despite the youngest patients being substantially sicker than their older counterparts, PHETI success was not decreased in this subgroup as it was in the Tarpgaard et al. study. Further details on GSA-HEMS training and materials are available in the forthcoming study described above [2]. Additionally, an *Annals* editorial in response to this same article, written by clinicians at a major American children’s hospital, summarizes some key aspects of the GSA-HEMS approach to optimising performance and safety of paediatric intubation [7].
